# Supplementary material for: Dual recognition of multiple signals in bacterial outer membrane proteins enhances assembly and maintains membrane integrity
Source: eLife. 2024 Jan 16;12:RP90274. doi: 10.7554/eLife.90274 (PMC10945584; doi:10.7554/eLife.90274)
Supplement: Supplementary file 1. [file elife-90274-supp1.docx]

**Supplemental FILE 1: Bacterial strains.**

| **Strain** | **Use** | **Ref/Source** |
| --- | --- | --- |
| BL21(DE3) | EMM isolation and *in vitro* studies. |  |
| BL21(DE3)* | Protein expression. | Invitrogen |
| MC4100A | *In vivo* studies, EMM isolation, and *in vitro* studies. |  |
| bamD-depletion | *In vivo* studies, EMM isolation, and *in vitro* studies. | This study |
| DH5α | Cloning strain. |  |
