# Supplementary material for: Dual recognition of multiple signals in bacterial outer membrane proteins enhances assembly and maintains membrane integrity
Source: eLife. 2024 Jan 16;12:RP90274. doi: 10.7554/eLife.90274 (PMC10945584; doi:10.7554/eLife.90274)
Supplement: Supplementary file 2. [file elife-90274-supp2.docx]

**upplemental FILE 2: Primers**

| **Primer name** | **Sequence 5'->3'** |
| --- | --- |
| pTnTl-f | ACTTAATACGACTCACTATAGGCTA |
| pTnT-r | GGATCCAAAAAACCCCTCAAGACCC |
| pSp64-f | ACCTTATGTATCATACACAT |
| pSp64-r | ACAGCTATGACATGATTACG |
| pTNTEspP-f | tgttctttttgcactcgagATGGATGTAACGCCGGTCATTAC |
| pTNTEspP-r | ccgcccgggtcgactctagaTCAGAACGAGTAACGGAAAT |
| pET15bOmpC-f | tgccgcgcggcagccatatgGAAGTTTACAACAAAGACGG |
| pET15bOmpC-r | ttagcagccggatcctcgagTTAGAACTGGTAAACCAGAC |
| pTnTOmpA-f | tgttctttttgcactcgagATGGCTCCGAAAGATAACACCTGGTACACTG |
| pTnTOmpA-r | ccgcccgggtcgactctagaTTAGAACTGGTAAACGATACCCACAGCAAC |
| pTnTOmpF-f | tgttctttttgcactcgagATGATGAAGCGCAATATTCTGGCAGTGATCG |
| pTnTOmpF-r | ccgcccgggtcgactctagaTTAGAACTGGTAAACGATACCCACAGCAACGGTGT |
| pTnTLamB-f | tgttctttttgcactcgagATGGTTGATTTCCACGGCTATGCACGTTCCGGTATTGGTTG |
| pTnTLamB-r | ccgcccgggtcgactctagaTTACCACCAGATTTCCATCTGGGCA |
| OmpCW77A-f | GACCGGTTACGGCCAGgcaGAATATCAGATCCAGG |
| OmpCW77A-r | CCTGGATCTGATATTCtgcCTGGCCGTAACCGGTC |
| OmpCE78A-f | CGGTTACGGCCAGTGGgcaTATCAGATCCAGGGCA |
| OmpCE78A-r | TGCCCTGGATCTGATAtgcCCACTGGCCGTAACCG |
| OmpCE78K-f | CGGTTACGGCCAGTGGaaaTATCAGATCCAGGGCA |
| OmpCE78K-r | TGCCCTGGATCTGATAtttCCACTGGCCGTAACCG |
| OmpCY79A-f | TTACGGCCAGTGGGAAgcaCAGATCCAGGGCAACA |
| OmpCY79A-r | TGTTGCCCTGGATCTGtgcTTCCCACTGGCCGTAA |
| OmpCK276A-f | CCTGGGTTGGGCGAACgcaGCACAGAACTTCGAAG |
| OmpCK276A-r | CTTCGAAGTTCTGTGCtgcGTTCGCCCAACCCAGG |
| OmpCK276E-f | CCTGGGTTGGGCGAACgaaGCACAGAACTTCGAAG |
| OmpCK276E-r | CTTCGAAGTTCTGTGCttcGTTCGCCCAACCCAGG |
| OmpCF280A-f | GAACAAAGCACAGAACgcaGAAGCTGTTGCTCAGT |
| OmpCF280A-r | ACTGAGCAACAGCTTCtgcGTTCTGTGCTTTGTTC |
| OmpCF280V-f | AACAAAGCACAGAACgtgGAAGCTGTTGCTCAG |
| OmpCF280V-r | CTGAGCAACAGCTTCcacGTTCTGTGCTTTGTT |
| OmpCE281A-f | CAAAGCACAGAACTTCgcaGCTGTTGCTCAGTACC |
| OmpCE281A-r | GGTACTGAGCAACAGCtgcGAAGTTCTGTGCTTTG |
| OmpCE281K-f | CAAAGCACAGAACTTCaaaGCTGTTGCTCAGTACC |
| OmpCE281K-r | GGTACTGAGCAACAGCtttGAAGTTCTGTGCTTTG |
| OmpCY286A-f | CGAAGCTGTTGCTCAGgcaCAGTTCGACTTCGGTC |
| OmpCY286A-r | GACCGAAGTCGAACTGtgcCTGAGCAACAGCTTCG |
| OmpCP294A-f | CGACTTCGGTCTGCGTgcaTCCCTGGCTTACCTGC |
| OmpCP294A-r | GCAGGTAAGCCAGGGAtgcACGCAGACCGAAGTCG |
| OmpCY325A-f | GATGTTGGTGCTACCgcgTACTTCAACAAAAAC |
| OmpCY325A-r | GTTTTTGTTGAAGTAcgcGGTAGCACCAACATC |
| OmpCY365A-f | AGCTCTGGGTCTGGTTgccCAGTTCTAAGTCGA |
| OmpCY365A-r | TCGACTTAGAACTGggcAACCAGACCCAGAGCT |
| OmpCBsigAAA-f | CTGGGTCTGGTTgccgcggcgTAAGTCGACCCGGGC |
| OmpCBsigAAA-r | GCCCGGGTCGACTTAcgccgcggcAACCAGACCCAG |
| OmpFV279A-f | AACAAAACGCAAGACgcgCTGTTAGTTGCGCAA |
| OmpFV279A-r | TTGCGCAACTAACAGcgcGTCTTGCGTTTTGTT |
| OmpFY285A-f | CTGTTAGTTGCGCAAgcgCAGTTCGATTTCGGT |
| OmpFY285A-r | ACCGAAATCGAACTGcgcTTGCGCAACTAACAG |
| LamBV333A-f | ACCAAGTGGTGGACCgcgGGTATTCGCCCGATG |
| LamBV333A-r | CATCGGGCGAATACCcgcGGTCCACCACTTGGT |
| LamBY339A-f | GGTATTCGCCCGATGgcgAAGTGGACGCCAATC |
| LamBY339A-r | GATTGGCGTCCACTTcgcCATCGGGCGAATACC |
| FLAGOmpC1-f | aacaggaggaattaaccATGAAAGTTAAAGTACTGTC |
| FLAGOmpC1-r | cttatcatcatcatccttataatcAGCAGCGTTTGCTGCGCCTG |
| FLAGOmpC2-f | gattataaggatgatgatgataagGAAGTTTACAACAAAGACGG |
| FLAGOmpC2-r | TACCAGCTGCAGATCTCGAGtcgacTTAGAACTGGTAAACCAGAC |
| BamDR49C-f | CAGGACGGTAACTGGtgcCAGGCAATAACGCAA |
| BamDR49C-r | TTGCGTTATTGCCTGgcaCCAGTTACCGTCCTG |
| BamDN60C-f | CTGGAAGCGTTAGATtgcCGCTATCCGTTTGGT |
| BamDN60C-r | ACCAAACGGATAGCGgcaATCTAACGCTTCCAG |
| BamDG65C-f | AATCGCTATCCGTTTtgcCCGTATTCGCAGCAG |
| BamDG65C-r | CTGCTGCGAATACGGgcaAAACGGATAGCGATT |
| BamDG200C-f | GTTAACCGCGTAGAAtgcATGTTGCGCGACTAC |
| BamDG200C-r | GTAGTCGCGCAACATgcaTTCTACGCGGTTAAC |
| BamDR203C-f | GTAGAAGGCATGTTGtgcGACTACCCGGATACC |
| BamDR203C-r | GGTATCCGGGTAGTCgcaCAACATGCCTTCTAC |
| BamDD204C-f | GAAGGCATGTTGCGCtgcTACCCGGATACCCAG |
| BamDD204C-r | GAAGGCATGTTGCGCtgcTACCCGGATACCCAG |
| OmpCQ278C-f | TGGGCGAACAAAGCAtgcAACTTCGAAGCTGTT |
| OmpCQ278C-r | AACAGCTTCGAAGTTgcaTGCTTTGTTCGCCCA |
| OmpCA282C-f | GCACAGAACTTCGAAtgcGTTGCTCAGTACCAG |
| OmpCA282C-r | CTGGTACTGAGCAACgcaTTCGAAGTTCTGTGC |
| OmpCA284C-f | AACTTCGAAGCTGTTtgcCAGTACCAGTTCGAC |
| OmpCA284C-r | GTCGAACTGGTACTGgcaAACAGCTTCGAAGTT |
| OmpCP294C-f | GACTTCGGTCTGCGTtgcTCCCTGGCTTACCTG |
| OmpCP294C-r | CAGGTAAGCCAGGGAgcaACGCAGACCGAAGTC |
| OmpCL296C-f | GGTCTGCGTCCGTCCtgcGCTTACCTGCAGTCT |
| OmpCL296C-r | AGACTGCAGGTAAGCgcaGGACGGACGCAGACC |
| OmpCY298C-f | CGTCCGTCCCTGGCTtgcCTGCAGTCTAAAGGT |
| OmpCY298C-r | ACCTTTAGACTGCAGgcaAGCCAGGGACGGACG |
| OmpCQ300C-f | TCCCTGGCTTACCTGtgcTCTAAAGGTAAAAAC |
| OmpCQ300C-r | GTTTTTACCTTTAGAgcaCAGGTAAGCCAGGGA |
| OmpCK302C-f | GCTTACCTGCAGTCTtgcGGTAAAAACCTGGGT |
| OmpCK302C-r | ACCCAGGTTTTTACCgcaAGACTGCAGGTAAGC |
| OmpCK317C-f | GACGAAGATATCCTGtgcTATGTTGATGTTGGT |
| OmpCK317C-r | ACCAACATCAACATAgcaCAGGATATCTTCGTC |
| OmpCV319C-f | GATATCCTGAAATATtgcGATGTTGGTGCTACC |
| OmpCV319C-r | GGTAGCACCAACATCgcaATATTTCAGGATATC |
| OmpCA323C-f | TATGTTGATGTTGGTtgcACCTACTACTTCAAC |
| OmpCA323C-r | GTTGAAGTAGTAGGTgcaACCAACATCAACATA |
| OmpCT333C-f | AACAAAAACATGTCCtgcTACGTTGACTACAAA |
| OmpCT333C-r | TTTGTAGTCAACGTAgcaGGACATGTTTTTGTT |
| OmpCV335C-f | AACATGTCCACCTACtgcGACTACAAAATCAAC |
| OmpCV335C-r | GTTGATTTTGTAGTCgcaGTAGGTGGACATGTT |
| OmpCN357C-f | GGCATCAACACTGATtgcATCGTAGCTCTGGGT |
| OmpCN357C-r | ACCCAGAGCTACGATgcaATCAGTGTTGATGCC |
| OmpCL363C-f | ATCGTAGCTCTGGGTtgcGTTTACCAGTTCTAA |
| OmpCL363C-r | TTAGAACTGGTAAACgcaACCCAGAGCTACGAT |
| OmpCF367C-f | GGTCTGGTTTACCAGtgcTAAGTCGACCCGGGC |
| OmpCF367C-r | GCCCGGGTCGACTTAgcaCTGGTAAACCAGACC |
