# Supplementary material for: Dual recognition of multiple signals in bacterial outer membrane proteins enhances assembly and maintains membrane integrity
Source: eLife. 2024 Jan 16;12:RP90274. doi: 10.7554/eLife.90274 (PMC10945584; doi:10.7554/eLife.90274)
Supplement: Supplementary file 3. [file elife-90274-supp3.docx]

**Supplementary FILE 3: Primers for BPA introduction into BamD**

| **X** | **BamD(X)ambFwd** | **BamD(X)ambRev** |
| --- | --- | --- |
| 27 | GGGGTCAAAGGAAGAAtagCCTGATAATCCGCCAA | TTGGCGGATTATCAGGctaTTCTTCCTTTGACCCC |
| 41 | TTACGCGACTGCACAAtagAAGCTGCAGGACGGTA | TACCGTCCTGCAGCTTctaTTGTGCAGTCGCGTAA |
| 43 | GACTGCACAACAAAAGtagCAGGACGGTAACTGGA | TCCAGTTACCGTCCTGctaCTTTTGTTGTGCAGTC |
| 49 | GCAGGACGGTAACTGGtagCAGGCAATAACGCAAC | GTTGCGTTATTGCCTGctaCCAGTTACCGTCCTGC |
| 53 | CTGGAGACAGGCAATAtagCAACTGGAAGCGTTAG | CTAACGCTTCCAGTTGctaTATTGCCTGTCTCCAG |
| 60 | ACTGGAAGCGTTAGATtagCGCTATCCGTTTGGTC | GACCAAACGGATAGCGctaATCTAACGCTTCCAGT |
| 62 | AGCGTTAGATAATCGCtagCCGTTTGGTCCGTATT | AATACGGACCAAACGGctaGCGATTATCTAACGCT |
| 64 | AGATAATCGCTATCCGtagGGTCCGTATTCGCAGC | GCTGCGAATACGGACCctaCGGATAGCGATTATCT |
| 65 | TAATCGCTATCCGTTTtagCCGTATTCGCAGCAGG | CCTGCTGCGAATACGGctaAAACGGATAGCGATTA |
| 70 | TGGTCCGTATTCGCAGtagGTGCAGCTGGATCTCA | TGAGATCCAGCTGCACctaCTGCGAATACGGACCA |
| 77 | GCAGCTGGATCTCATCtagGCCTACTATAAAAACG | CGTTTTTATAGTAGGCctaGATGAGATCCAGCTGC |
| 79 | GGATCTCATCTACGCCtagTATAAAAACGCCGATT | AATCGGCGTTTTTATActaGGCGTAGATGAGATCC |
| 89 | CGATTTGCCGTTAGCAtagGCTGCCATCGATCGTT | AACGATCGATGGCAGCctaTGCTAACGGCAAATCG |
| 98 | CGATCGTTTTATTCGCtagAACCCGACCCATCCGA | TCGGATGGGTCGGGTTctaGCGAATAAAACGATCG |
| 101 | TATTCGCCTTAACCCGtagCATCCGAATATCGATT | AATCGATATTCGGATGctaCGGGTTAAGGCGAATA |
| 105 | CCCGACCCATCCGAATtagGATTATGTCATGTACA | TGTACATGACATAATCctaATTCGGATGGGTCGGG |
| 109 | GAATATCGATTATGTCtagTACATGCGTGGCCTGA | TCAGGCCACGCATGTActaGACATAATCGATATTC |
| 114 | CATGTACATGCGTGGCtagACCAATATGGCGCTGG | CCAGCGCCATATTGGTctaGCCACGCATGTACATG |
| 117 | GCGTGGCCTGACCAATtagGCGCTGGATGACAGTG | CACTGTCATCCAGCGCctaATTGGTCAGGCCACGC |
| 121 | CAATATGGCGCTGGATtagAGTGCGCTGCAAGGGT | ACCCTTGCAGCGCACTctaATCCAGCGCCATATTG |
| 124 | GCTGGATGACAGTGCGtagCAAGGGTTCTTTGGCG | CGCCAAAGAACCCTTGctaCGCACTGTCATCCAGC |
| 136 | CGATCGTAGCGATCGCtagCCTCAACATGCACGAG | CTCGTGCATGTTGAGGctaGCGATCGCTACGATCG |
| 138 | TAGCGATCGCGATCCTtagCATGCACGAGCTGCGT | ACGCAGCTCGTGCATGctaAGGATCGCGATCGCTA |
| 148 | TGCGTTTAGTGACTTTtagAAACTGGTGCGCGGCT | AGCCGCGCACCAGTTTctaAAAGTCACTAAACGCA |
| 149 | GTTTAGTGACTTTTCCtagCTGGTGCGCGGCTATC | GATAGCCGCGCACCAGctaGGAAAAGTCACTAAAC |
| 153 | TTCCAAACTGGTGCGCtagTATCCGAACAGTCAGT | ACTGACTGTTCGGATActaGCGCACCAGTTTGGAA |
| 158 | CGGCTATCCGAACAGTtagTACACCACCGATGCCA | TGGCATCGGTGGTGTActaACTGTTCGGATAGCCG |
| 164 | GTACACCACCGATGCCtagAAACGTCTGGTATTCC | GGAATACCAGACGTTTctaGGCATCGGTGGTGTAC |
| 168 | TGCCACCAAACGTCTGtagTTCCTGAAAGATCGTC | GACGATCTTTCAGGAActaCAGACGTTTGGTGGCA |
| 176 | GAAAGATCGTCTGGCGtagTATGAATACTCCGTGG | CCACGGAGTATTCATActaCGCCAGACGATCTTTC |
| 181 | GAAATATGAATACTCCtagGCCGAGTACTATACAG | CTGTATAGTACTCGGCctaGGAGTATTCATATTTC |
| 184 | ATACTCCGTGGCCGAGtagTATACAGAACGTGGCG | CGCCACGTTCTGTATActaCTCGGCCACGGAGTAT |
| 186 | CGTGGCCGAGTACTATtagGAACGTGGCGCATGGG | CCCATGCGCCACGTTCctaATAGTACTCGGCCACG |
| 192 | AGAACGTGGCGCATGGtagGCCGTCGTTAACCGCG | CGCGGTTAACGACGGCctaCCATGCGCCACGTTCT |
| 196 | ATGGGTTGCCGTCGTTtagCGCGTAGAAGGCATGT | ACATGCCTTCTACGCGctaAACGACGGCAACCCAT |
| 200 | CGTTAACCGCGTAGAAtagATGTTGCGCGACTACC | GGTAGTCGCGCAACATctaTTCTACGCGGTTAACG |
| 204 | AGAAGGCATGTTGCGCtagTACCCGGATACCCAGG | CCTGGGTATCCGGGTActaGCGCAACATGCCTTCT |
| 232 | GATGAATGCGCAAGCTtagAAAGTAGCGAAAATCA | TGATTTTCGCTACTTTctaAGCTTGCGCATTCATC |
| 233 | GAATGCGCAAGCTGAAtagGTAGCGAAAATCATCG | CGATGATTTTCGCTACctaTTCAGCTTGCGCATTC |
| 237 | TGAAAAAGTAGCGAAAtagATCGCCGCAAACAGCA | TGCTGTTTGCGGCGATctaTTTCGCTACTTTTTCA |
