# Supplementary material for: Dual recognition of multiple signals in bacterial outer membrane proteins enhances assembly and maintains membrane integrity
Source: eLife. 2024 Jan 16;12:RP90274. doi: 10.7554/eLife.90274 (PMC10945584; doi:10.7554/eLife.90274)
Supplement: Supplementary file 4. [file elife-90274-supp4.docx]

**Supplementary FILE 4: Plasmids for *in vitro* translation.**

| **Plasmid name** | **Synthesized protein** | **Vector** | **Primers for construct** | **RE Site** | **Template DNA, source, or method** |
| --- | --- | --- | --- | --- | --- |
| pTnT-EspP | EspP | pTnT | pTNTEspP-f / pTNTEspP-r | XhoI/XbaI | gblock,  SLiCE |
| pTnT-OmpA | OmpA | pTnT | pTnTOmpA-f / pTnTOmpA-r | XhoI/XbaI | K-12 gene,  SLiCE |
| pTnT-OmpC | OmpC | pTnT | -- | XhoI/XbaI | (Gunasinghe et al., 2018) |
| pTNT-OmpF | OmpF | pTnT | pTnTOmpF-f / pTnTOmpF-r | XhoI/XbaI | K-12 gene, SLiCE |
| pTnT-LamB | LamB | pTnT | pTnTLamB-f / pTnTLamB-r | XhoI/XbaI | K-12 gene,  SLiCE |
| pTnT-OmpC-W77A | OmpC W77A | pTnT | OmpCW77A-f / OmpCW77A-r |  | Quick change mutagenesis |
| pTnT-OmpC-E78A | OmpC E78A | pTnT | OmpCE78A-f / OmpCE78A-r |  | Quick change mutagenesis |
| pTnT-OmpC-E78K | OmpC E78K | pTnT | OmpCE78K-f / OmpCE78K-r |  | Quick change mutagenesis |
| pTnT-OmpC-Y79A | OmpC Y79A | pTnT | OmpCY79A-f / OmpCY79A-r |  | Quick change mutagenesis |
| pTnT-OmpC-K276E | OmpC K276E | pTnT | OmpCK276E-f / OmpCK276E-r |  | Quick change mutagenesis |
| pTnT-OmpC-F276A | OmpC F276A | pTnT | OmpCK276A-f / OmpCK276A-r |  | Quick change mutagenesis |
| pTnT-OmpC-F280A | OmpC F280A | pTnT | OmpCF280A-f / OmpCF280A-r |  | Quick change mutagenesis |
| pTnT-OmpC-F280V | OmpC F280V | pTnT | OmpCF280V-f / OmpCF280V-r |  | Quick change mutagenesis |
| pTnT-OmpC-E281A | OmpC E281A | pTnT | OmpCE281A-f / OmpCE281A-r |  | Quick change mutagenesis |
| pTnT-OmpC-E281K | OmpC E281K | pTnT | OmpCE281K-f / OmpCE281K-r |  | Quick change mutagenesis |
| pTnT-OmpC-Y286A | OmpC Y286A | pTnT | OmpCY286A-f / OmpCY286A-r |  | Quick change mutagenesis |
| pTnT-OmpC-P294A | OmpC P294A | pTnT | OmpCP294A-f / OmpCP294A-r |  | Quick change mutagenesis |
| pTnT-OmpC-Y325A | OmpC Y325A | pTnT | OmpCY325A-f / OmpCY325A-r |  | Quick change mutagenesis |
| pTnT-OmpC-Y365A | OmpC Y365A | pTnT | OmpCY365A-f / OmpCY365A-r |  | Quick change mutagenesis |
| pTnT-OmpC-b-sigAAA | OmpC G352A, L354A, F359A | pTnT | OmpCBsigAAA-f / OmpCBsigAAA-r |  | Quick change mutagenesis |
| pTnT-OmpC-A284C,L296C | OmpC A284C, L296C | pTnT | OmpCA284C-f/r, OmpCL296C-f/r |  | Quick change mutagenesis |
| pTnT-OmpC-A323C, V335C | OmpC A323C, V335C | pTnT | OmpCA323C-f/r, OmpCV335C-f/r |  | Quick change mutagenesis |
| pTnT-OmpC-A323C, V335C | OmpC A323C, V335C | pTnT | OmpCA323C-f/r, OmpCV335C-f/r |  | Quick change mutagenesis |
| pTnt-OmpC-T333C, L363C | OmpC T333C, L363C | pTnT | OmpCT333C-f/r, OmpCL363C-f/r |  |  |
| pTnT-OmpF-V279A | OmpF V279A | pTnT | OmpFV279A-f / OmpFV279A-r |  | Quick change mutagenesis |
| pTnT-OmpF-Y285A | OmpF Y285A | pTnT | OmpFY285A-f / OmpFY285A-r |  | Quick change mutagenesis |
| pTnT-LamB-V333A | LamB V333A | pTnT | LamBV333A-f / LamBV333A-r |  | Quick change mutagenesis |
| pTnT-LamB-Y339A | LamB Y339A | pTnT | LamBY339A-f / LamBY339A-r |  | Quick change mutagenesis |
