# Supplementary material for: Dual recognition of multiple signals in bacterial outer membrane proteins enhances assembly and maintains membrane integrity
Source: eLife. 2024 Jan 16;12:RP90274. doi: 10.7554/eLife.90274 (PMC10945584; doi:10.7554/eLife.90274)
Supplement: Supplementary file 5. [file elife-90274-supp5.docx]

**Supplementary FILE 5: Plasmids for recombinant protein expression.**

| **Plasmid name** | **Expressed protein** | **Vector/Promoter** | **Primers for construct** | **RE site** | **Template DNA, source, or method** |
| --- | --- | --- | --- | --- | --- |
| pET22-BamAL4H8 | BamA His8 at loop4 | pET22b/ T7 | -- |  | (Ding et al., 2020) |
| pHIS-BamB | His6-TEVsite-BamB | pHIS2-parallel/ T7 | -- |  | (Chen et al., 2021) |
| pHIS-BamC | His6-TEVsite-BamC | pHIS2-parallel/ T7 | -- |  | (Chen et al., 2021) |
| pHIS-BamD | His6-TEVsite-BamD | pHIS2-parallel/ T7 | -- |  | (Chen et al., 2021) |
| pET15b-OmpC | His6-TEVsite-OmpC | pET15b/ T7 | pTnTOmpC-f / pTnTOmpC-r | XhoI/XbaI | K-12 gene,  SLiCE |
| pET15b-OmpC Y286A | His6-TEVsite-BamD | pET15b/ T7 | OmpCY286A-f / OmpCY286A-r |  | Quick change mutagenesis |
| pBAD-FLOmpC | FLAG-OmpC-FLAG | pBAD/ areBAD | FLAGOmpC1-f /FLAGOmpC1-r, FLAGOmpC2-f /FLAGOmpC2-r |  | K-12 gene,  SLiCE |
| pBAD-FLOmpC F280A | FLAG-OmpC F280A | pBAD/ areBAD | OmpCF280A-f / OmpCF280A-r |  | pBAD-FLOmpC,  Quick change mutagenesis |
| pBAD-FLOmpC Y286A | FLAG-OmpC Y286A | pBAD/ areBAD | OmpCY286A-f / OmpCY286A-r |  | pBAD-FLOmpC,  Quick change mutagenesis |
| pBAD-FLOmpC F280A Y286A | FLAG-OmpC F280A Y286A | pBAD/ areBAD | OmpCY286A-f / OmpCY286A-r |  | pBAD-FLOmpC F280A  Quick change mutagenesis |
| pBAD-FLOmpC-VY | OmpC-FLAG V359A, Y365A | pBAD/ araBAD | OmpCV359A-f/r, OmpCY365A-f/r |  | pBAD-FLOmpC, quick change mutagenesis |
| pHIS-BamD (X) amber | His6-TEVsite-BamD (X) BPA | pBAD/ areBAD | BamD(X)ambFwd/ BamD(X)ambRev |  | pHIS-BamD,  Quick change mutagenesis |
