# Supplementary material for: Dual recognition of multiple signals in bacterial outer membrane proteins enhances assembly and maintains membrane integrity
Source: eLife. 2024 Jan 16;12:RP90274. doi: 10.7554/eLife.90274 (PMC10945584; doi:10.7554/eLife.90274)
Supplement: Supplementary file 6. [file elife-90274-supp6.docx]

**Supplementary FILE 6: Plasmids for *in vivo* protein expression.**

| **Plasmid name** | **Expressed protein** | **Vector/Promoter** | **Primers for construct** | **RE site** | **Template DNA, source, or method** |
| --- | --- | --- | --- | --- | --- |
| pAp-BamD | BamD | pTnT/ BamA | BamDSL-f / BamDSL-r | XbaI/SalI | K-12 gene |
| pAp-BamD-His8 | BamD-His8 | pTnT/ BamA | BamDSL-f / BamDCHis8SL-r | XbaI/SalI | K-12 gene |
| pAp-BamD-His8 Y62A | BamD-His8 Y62A | pTnT/ BamA | BamDY62A-f / BamDY62A-r |  | pAp-BamD-His8, Quick change mutagenesis |
| pAp-BamD-His8 R197A | BamD-His8 R197A | pTnT/ BamA | BamDR197A-f / BamDR197A-r |  | pAp-BamD-His8, Quick change mutagenesis |
