# Supplementary material for: Dual recognition of multiple signals in bacterial outer membrane proteins enhances assembly and maintains membrane integrity
Source: eLife. 2024 Jan 16;12:RP90274. doi: 10.7554/eLife.90274 (PMC10945584; doi:10.7554/eLife.90274)
Supplement: Supplementary file 7. [file elife-90274-supp7.docx]

**Supplementary FILE 7: Structural Data of this study**

| **Protein Name** | **PDB ID** | **PDB DOI** | **Citation** |
| --- | --- | --- | --- |
| OmpX | 1QJ8 | 10.2210/pdb1QJ8/pdb | (Vogt and Schulz, 1999) |
| OmpA | 1BXW | 10.2210/pdb1BXW/pdb | (Pautsch and Schulz, 1998) |
| OmpW | 2F1T | 10.2210/pdb2F1T/pdb | (Hong et al., 2006) |
| PagP | 3GP6 | 10.2210/pdb3GP6/pdb | (Cuesta-Seijo et al., 2010) |
| OmpT | 1I78 | 10.2210/pdb1I78/pdb | (Vandeputte-Rutten et al., 2001) |
| EspP | 2QOM | 10.2210/pdb2QOM/pdb | (Barnard et al., 2007) |
| HBP | 3AEH | 10.2210/pdb3AEH/pdb | (Tajima et al., 2010) |
| NanC | 2WJQ | 10.2210/pdb2WJQ/pdb | (Wirth et al., 2009) |
| PldA/OmpLA | 1QD5 | 10.2210/pdb1QD5/pdb | (Snijder et al., 1999) |
| Tsx | 1TLW | 10.2210/pdb1TLW/pdb | (Ye and van den Berg, 2004) |
| FadL | 1T16 | 10.2210/pdb1T16/pdb | (van den Berg et al., 2004) |
| OmpC | 2J1N | 10.2210/pdb2J1N/pdb | (Baslé et al., 2006) |
| PhoE | 1PHO | 10.2210/pdb1PHO/pdb | (Cowan et al., 1992) |
| OmpF | 1BT9 | 10.2210/pdb1BT9/pdb | (Phale et al., 1998) |
| LamB | 1MPM | 10.2210/pdb1MPM/pdb | (Dutzler et al., 1996) |
| BtuB | 1NQE | 10.2210/pdb1NQE/pdb | (Chimento et al., 2003) |
| CirA | 2HDI | 10.2210/pdb2HDI/pdb | (Buchanan et al., 2007) |
| FhuA | 1BY3 | 10.2210/pdb1BY3/pdb | (Locher et al., 1998) |
| FepA | 1FEP | 10.2210/pdb1FEP/pdb | (Buchanan et al., 1999) |
| FecA | 1KMO | 10.2210/pdb1KMO/pdb | (Ferguson et al., 2002) |
| PapC | 2VQI | 10.2210/pdb2VQI/pdb | (Remaut et al., 2008) |
