# Supplementary material for: Dual recognition of multiple signals in bacterial outer membrane proteins enhances assembly and maintains membrane integrity
Source: eLife. 2024 Jan 16;12:RP90274. doi: 10.7554/eLife.90274 (PMC10945584; doi:10.7554/eLife.90274)
Supplement: Supplementary file 8. [file elife-90274-supp8.docx]

**Supplementary FILE 8: Characterization of BamA molecules in the membrane layer**

|  | | | | | | | | |
| --- | --- | --- | --- | --- | --- | --- | --- | --- |
| Layer | t (Å) | SLD (🞨10^-6^ Å^-2^) | | | Φ (%) | | | σ (Å) |
|  |  | D_2_O | GMW5 | H_2_O | BamA | POPC | Solution |  |
| Cr | 79.7±0.8 | 3.03 | 3.03 | 3.00 | - | - | - | 12.1±0.2 |
| Au | 251.8±5.1 | 3.88 | 3.79 | 3.40 | - | - | - | 13.0±0.1 |
| NTA | 8.0±0.7 | 5.00 | 4.28 | 0.43 | - | - | 66.5±3.5 | 10.0±0.1 |
| His_6_ | 6.0±0.5 | 4.94 | 4.46 | 2.50 | - | - | 25.3±7.4 | 4.0±0.1 |
| *β*-Barrel | 56.9±1.5 | 3.88 | 2.93 | 0.18 | 17.3±0.6 | 35.1±1.6 | 47.6±2.9 | 10.1±0.1 |
| P3-5 | 29.5±0.8 | 5.96 | 4.48 | -0.29 | 10.7±0.9 | 3.4±1.7 | 85.9±2.6 | 7.0±0.1 |
| P1-2 | 31.2±0.8 | 6.14 | 4.73 | -0.30 | 11.2±0.8 | - | 90.0±2.5 | 13.8±0.1 |
| t: thickness; SLD: scattering length density; Φ: volume fraction; σ: roughness; P3-5: POTRA3, POTRA4 and POTRA5; P1-2: POTRA1 and POTRA 2. | | | | | | | | |
