# Supplementary material for: Dual recognition of multiple signals in bacterial outer membrane proteins enhances assembly and maintains membrane integrity
Source: eLife. 2024 Jan 16;12:RP90274. doi: 10.7554/eLife.90274 (PMC10945584; doi:10.7554/eLife.90274)
Supplement: Supplementary file 9. [file elife-90274-supp9.docx]

**Supplementary FILE 9: Characterization of BamAD molecules in the membrane layer**

|  | | | | | | | | | |
| --- | --- | --- | --- | --- | --- | --- | --- | --- | --- |
| Layer | t (Å) | SLD (🞨10^-6^ Å^-2^) | | | Φ (%) | | | | σ (Å) |
|  |  | D_2_O | GMW5 | H_2_O | BamA | POPC | BamD | Solution |  |
| Cr | 78.5±2.6 | 3.09 | 3.01 | 3.00 | - | - | - | - | 10.0±0.1 |
| Au | 254.6±3.6 | 3.88 | 3.87 | 3.60 | - | - | - | - | 13.1±0.1 |
| NTA | 9.6±1.0 | 5.24 | 4.05 | 0.46 | - | - | - | 64.8±5.9 | 10.1±0.1 |
| His_6_ | 7.1±0.8 | 5.00 | 4.44 | 2.50 | - | - | - | 25.8±7.0 | 4.0±0.1 |
| *β*-Barrel | 58.7±0.4 | 3.94 | 2.56 | 0.18 | 18.4±1.1 | 35.4±5.5 | - | 46.2±6.7 | 12.6±1.8 |
| P3-5 | 29.7±0.8 | 5.41 | 4.36 | -0.11 | 11.0±0.8 | 3.3±0.5 | 8.8±1.1 | 76.9±2.0 | 6.1±0.1 |
| P1-2 | 30.3±0.4 | 6.09 | 4.66 | -0.30 | 11.2±2.9 | - | - | 88.8±2.9 | 13.7±0.1 |
| t: thickness; SLD: scattering length density; Φ: volume fraction; σ: roughness; P3-5: POTRA3, POTRA4 and POTRA5; P1-2: POTRA1 and POTRA 2. | | | | | | | | | |
