# Supplementary material for: Dual recognition of multiple signals in bacterial outer membrane proteins enhances assembly and maintains membrane integrity
Source: eLife. 2024 Jan 16;12:RP90274. doi: 10.7554/eLife.90274 (PMC10945584; doi:10.7554/eLife.90274)
Supplement: Supplementary file 10. [file elife-90274-supp10.docx]

**Supplementary FILE 10: Characterization of BamAD-OmpC molecules in the membrane layer**

|  | | | | | | | | | | |
| --- | --- | --- | --- | --- | --- | --- | --- | --- | --- | --- |
| Layers*^a^* | t (Å) | SLD (🞨10^-6^ Å^-2^) | | | Φ (%) | | | | | σ (Å) |
|  |  | D_2_O | GMW5.3 | H_2_O | BamA | POPC | BamD | OmpC | Solution |  |
| Cr | 79.8±3.4 | 3.08 | 3.07 | 3.02 | - | - | - | - | - | 16.6±2.4 |
| Au | 253.0±5.6 | 3.85 | 3.72 | 3.69 | - | - | - | - | - | 15.3±1.7 |
| NTA | 9.8±1.5 | 5.28 | 4.20 | 0.45 | - | - | - | - | 68.3±2.4 | 9.9±0.1 |
| His_6_ | 9.4±0.5 | 4.86 | 4.37 | 2.31 | - | - | - | - | 22.7±1.7 | 4.0±0.1 |
| *β*-Barrel | 56.8±1.5 | 3.78 | 2.82 | 0.22 | 18.3±1.2 | 35.9±1.5 | - | - | 45.8±2.7 | 6.0±0.1 |
| P3-5 | 42.5±1.6 | 5.81 | 4.54 | -0.09 | 11.7±2.9 | 3.3±0.9 | 9.0±3.3 | 8.0±1.2 | 76.0±7.3 | 12.1±0.1 |
| P1-2 | 28.5±0.9 | 6.09 | 4.82 | -0.28 | 10.7±0.9 | - | - | - | 89.3±0.9 | 5.0±0.1 |
| t: thickness; SLD: scattering length density; Φ: volume fraction; σ: roughness; P3-5: POTRA3, POTRA4 and POTRA5; P1-2: POTRA1 and POTRA 2. | | | | | | | | | | |
